# Immunologic Pathophysiology and Airway Remodeling Mechanism in Severe Asthma: Focused on IgE-Mediated Pathways

**DOI:** 10.3390/diagnostics11010083

**Published:** 2021-01-06

**Authors:** Shih-Lung Cheng

**Affiliations:** Department of Internal Medicine, Far Eastern Memorial Taipei Hospital, Department of Chemical Engineering and Materials Science, Yuan Ze University, Zhongli, Taoyuan 32056, Taiwan; shihlungcheng@gmail.com; Tel.: +886-(2)-89667000; Fax: +886-(2)-77380708

**Keywords:** asthma, IgE, airway remodeling

## Abstract

Despite the expansion of the understanding in asthma pathophysiology and the continual advances in disease management, a small subgroup of patients remains partially controlled or refractory to standard treatments. Upon the identification of immunoglobulin E (IgE) and other inflammatory mediators, investigations and developments of targeted agents have thrived. Omalizumab is a humanized monoclonal antibody that specifically targets the circulating IgE, which in turn impedes and reduces subsequent releases of the proinflammatory mediators. In the past decade, omalizumab has been proven to be efficacious and well-tolerated in the treatment of moderate-to-severe asthma in both trials and real-life studies, most notably in reducing exacerbation rates and corticosteroid use. While growing evidence has demonstrated that omalizumab may be potentially beneficial in treating other allergic diseases, its indication remains confined to treating severe allergic asthma and chronic idiopathic urticaria. Future efforts may be bestowed on determining the optimal length of omalizumab treatment, seeking biomarkers that could better predict treatment response and as well as extending its indications.

## 1. Introduction

Asthma is responsible for considerable global morbidity and healthcare costs [1]. It is a chronic respiratory inflammatory disease, which is characterized by shortness of breath, cough, wheezing and chest tightness. Severe asthma affects 5–10% of total asthma patients, but it accounts for over 50% of total costs [2]. There are two major phenotypes of severe asthma, which are severe allergic asthma (SAA) and severe eosinophilic asthma (SEA). The endotype of SAA and SEA belongs to type 2 (T2) inflammation, which consists of Th2 cells and innate lymphoid cell type 2 (ILC 2) cells. From Th2 predominant asthma point of view, allergic asthma is the most prevalent phenotype. In patients with allergic asthma, IgE is synthesized in lymph nodes and airway mucosa by lymphocytes B upon IL-4-induced Ig class switching, consisting of complex antibody recombination resulting in the prevalent production of IgE [3,4]. Moreover, IgE plays an important role from sensitization to the chronic phase in the allergic inflammatory pathway. IgE receptors can be divided into two types, high-affinity IgE receptors (FcεRI) and low-affinity IgE receptors (FcεRII) [5]. The biologic functions of IgE depend on the binding of two Cε3 domains to FcεRI and FcεRII located in several target cells. The underlying immunopathological mechanisms of asthma lead to chronic airway inflammation resulting in airway remodeling (AR), a process of structural changes of airway walls. In airway diseases, remodeling is associated with clinical outcomes such as more severe airflow obstruction and airway hyperresponsiveness [6,7]. In clinical practice, there are several biologics in the market to treat different phenotypes of severe asthma. Omalizumab, anti-IgE therapy, is the first biological therapy in asthma therapeutically treated area. After almost two decades of clinical practice, there are several references, which discussed the effects of anti-airway remodeling by omalizumab, have been published. At the same time, methods of airway remodeling evaluation are developing and growing. This review discusses the role of IgE in airway remodeling and evidence regarding anti-airway remodeling through anti-IgE treatment. Moreover, we discuss current methods of airway remodeling assessment and assist the clinician in understanding the clinical benefits of their uses.

## 2. The Role of IgE in Pathophysiology of Airway Remodeling in Asthma

Persistent inflammation of the airways not only causes asthma symptoms but also leads to the remodeling process. AR is a process of reconstruction of the bronchial wall, and it is characterized by (1) smooth muscle hypertrophy/ hyperplasia, (2) mucus gland hyperplasia, (3) shedding and metaplasia of the epithelium, (4) angiogenesis, (5) subepithelial collagen and glycoprotein deposition and (6) extracellular matrix (ECM) deposition in the submucosa, muscle and adventitia [8,9].

According to studies, allergic asthma is the predominant phenotype, which is over 60% of the total asthma group [10,11,12]. In addition, in allergic asthma, type 2 inflammatory pathway, including Th2 and ILC2, plays a major role in activating the immune pathway, which is mediated by related effective cells such as mast cells and eosinophils. Immunoglobulin E (IgE) has a central role in the pathobiology of allergic asthma [5]. There are two types of IgE: one is classical IgE, which is defined that allergen binds to IgE on IgE receptors on related inflammatory cells (e.g., mast cells), causing the release of proinflammatory mediators. The other one is cytokinergic IgE, which is a mechanism produced in the absence of allergen; IgE produced by IgE-class switched B cells activate the IgE receptor and is further propagated by interleukins. In this pathway, allergen avoidance is not effective [13]. The pleiotropic effects of IgE are mediated by activation of specific IgE receptors expressed by both immune-inflammatory (mast cells, eosinophils, basophils and dendritic cells) and airway structural cells (airway epithelium cells and airway smooth muscle cells (ASMCs)) [14,15,16,17,18].

Recent data have suggested that allergen exposure markedly increased the expression of immunoreactivity for three alarmin cytokine, TSLP, IL-22 and IL-25, in both human airway epithelium and bronchial mucosa [19]. Combining documented results and expression of IgE receptors on airway epithelium cells may indicate that IgE could directly interact with IgE receptors on airway epithelium cells, causing airway epithelium damage as well as leading to the release of alarmin to produce following inflammatory and remodeling process. In terms of ASMCs, IgE receptors are expressed by ASMCs as well [20,21,22]. Recent studies suggested that ASMCs responded to both IgE and cytokinergic IgE by increased proliferation, ECM production and collagen deposition [21,22,23]. In addition, the signaling pathway of ASMCs remodeling, which is activated by IgE, was also demonstrated. As reviewed by Kim et al. [24], the signal pathway activated by allergen-bonded IgE consist of phosphatidylinositol 3-kinases (PI3K) -> protein kinase B (Akt) -> mammalian target of rapamycin (mTOR) signaling. Moreover, Fang et al. [25] also indicated that Non-immune IgE-stimulated ASMCs remodeling by not only PI3K activation but also upregulating microRNA-21-5-p, which downregulated phosphatase and tensin homolog (PTEN) and enhanced mTOR signaling. Other AR mediators include Th2 cytokines (IL-4, IL-5, IL-9, IL-13), IL-6, transforming growth factor β (TGF-β), Endothelin-1 (ET-1), metalloproteinase-9 (MMP-9), prostaglandin D2 (PGD2), tumor necrosis factor-α (TNF-α) and others [26,27].

As a result of AR, asthma patients, who have persistent chronic airway inflammation, may experience airway obstruction, which leads to worsening lung function, symptoms and response to bronchodilators. Moreover, IgE receptors are expressed on several effective cells and structure cells; IgE, therefore, could directly interact with these inflammatory cells and leads to related cytokines, which all promote AR (Table 1).

### The Role of Omalizumab in Airway Remodeling in Asthma

Omalizumab, an anti-IgE treatment, was the first humanized biologic for SAA in the world. In addition, the mechanism of action of omalizumab are: (1) neutralization of free-form IgE and (2) downregulation of FcεRI on inflammatory cells (dendritic cells, eosinophils, and mast cells) as well as structure cells (epithelial cells and airway smooth muscle cells). Therefore, omalizumab could cover up- and downstream inflammatory processes through previous mechanisms [17]. Recent studies have found direct associations between IgE or anti-IgE treatment with features of airway remodeling. From a structure improvement perspective: Riccio et al. [28] showed that after 1 year of omalizumab treatment in severe allergic asthmatics, there was a significant reduction in reticular basement membrane (RBM) thickness and eosinophil infiltration in bronchial biopsies. Hoshino et al. published a study on the effects of omalizumab on anti-airway remodeling. In severe allergic asthmatics, 16-weeks of treatment with omalizumab decreased WA/BSA, WA percentage, and T/√BSA and increased Ai/BSA as assessed by CT. Omalizumab decreased the percentage of sputum eosinophils and increased FEV_1_ and AQLQ scores. Changes in FEV_1_ and sputum eosinophils correlated with changes in WA percentage [29]. Mauri et al. [30] showed that in severe allergic asthmatics, 1-year omalizumab treatment downregulated bronchial smooth muscle proteins. Among ECM proteins, galectin-3 correlated best with airway remodeling modulation by omalizumab. Tajiri et al. [31] reported that in severe allergic asthmatics, 48-weeks of treatment with omalizumab reduced 48% of WA percentage and thickness and increased Ai and Ai/BSA as assessed by CT. WA percentage changes significantly correlated with the decrease in FeNO_50_ levels and sputum eosinophils. Riccio et al. [32] showed that after 36 months of treatment with omalizumab, omalizumab-treatment responders could benefit from disease-modifying effects regarding the reduction of RBM thickness, bronchial eosinophilic infiltration, periostin level and airway smooth muscle proteins. Moreover, data also suggested that galactin-3 acts as a biomarker of modulation of AR upon treatment with omalizumab. Zastrzezynska et al. [33] reported that omalizumab might also decrease unfavorable structural airway changes in allergic asthmatics, including decreasing the fibronectin deposit and thickness of the basal lamina as well as reduction of the eosinophil counts in blood, bronchoalveolar lavage (BAL) and bronchial mucosa (Table 2).

For regulation of AR mediators perspective: It has been reported that omalizumab was able to significantly decrease eosinophil counts in blood, sputum and bronchial submucosa [34,35,36]. In terms of alarmin, Huang et al. [37] showed that the characteristics of omalizumab-responders are increasing alarmin in mRNA, protein level and immunohistochemical staining. Furthermore, omalizumab significantly decreased mRNA level of IL-33, IL-25 and TSLP with asthma control and lung function improvement. Zietkowski et al. [35] reported that omalizumab therapy in severe persistent allergic asthma patients resulted in decreased expression of ET-1 in the airways. This result also indicated that omalizumab could be limiting airway inflammation and bronchial structural changes such as subepithelial fibrosis and proliferation of ASMCs in patients with severe allergic asthma. Alongside this, other studies also reported that omalizumab significantly attenuated the production of TGF-β by bronchial epithelial cells [38] as well as induced a reduction effect on MMP-9 serum level [39] (Table 2).

From a lung function improvement perspective, a significant benefit regarding the change from baseline in FEV1% predicted for subcutaneous omalizumab versus placebo was observed by Normansell et al. [40]. In two real-world meta-analysis, the data demonstrated that lung function (FEV1% predicted) improved consistently from baseline by an average of 8.5% to 12.4% between 5 and 32 months, and by an average of 26% from baseline to ≥36 months [41,42]. Moreover, according to 1 post hoc analysis of 3 published randomized studies, data suggested that omalizumab may confer some protection to minimize lung function decline in children, adolescents and adults who experience asthma exacerbations while on therapy [43] (Table 2).

## 3. Current Methods of Airway Remodeling Assessment

Evaluation of airway remodeling could be divided into two parts: one is functional assessment, pulmonary function test, the other one is structural assessment. In this section, we summarize current methods of airway remodeling assessment. Methods for evaluating airway remodeling include: (1) direct assessment of airway tissues, (2) indirect assessment of using body fluids, (3) radiological assessment, and (4) physiological assessment [45].

## 4. Direct Airway Tissue Assessment

Direct airway tissue assessment is the most confident and strongest evidence regarding airway remodeling assessment. Surgical lung specimen: It can provide a unique and global view of pathological features by taking specimens from the central airway, alveoli, and pulmonary circulation. In addition, it can allow physicians to observe the changes in goblet cells, mucus gland, smooth muscle mass, subepithelial fibrosis, epithelial alteration, and angiogenesis. On the other hand, it also has limitations, including samples, which are difficult to obtain, less accurate knowledge of past medical history, inability to perform physiological tests, as well as few patients would be willing to undergo such invasive biopsy before and after treatment [45].

Endobronchial biopsy—performed under flexible bronchoscopy and is the standard approach to evaluating airway remodeling [46,47]. This procedure is the minimally invasive way to obtain central airway specimens (from carina/second carina) to determine changes to the central airway after treatment, including sub-epithelial thickening and smooth muscle mass. Limitations include that it does not allow the study of the remodeling of the entire airway, only the large airways. It also may lead to possible bleeding and pneumothorax complications, but less than with transbronchial biopsy [45].

Transbronchial biopsy—like endobronchial biopsy, it is also performed under flexible bronchoscopy. It can obtain specimens from the distal lung to allow the study of changes in the distal airway wall and alveolar tissues. Moreover, it is able to evaluate airway inflammation, and remodeling is comparable to surgical biopsy. However, to date, there are only a few studies which have been performed using transbronchial tissue due to high-risk of major bleeding, pneumothorax complications as well as low success rate to obtain adequate distal airway wall sample [45,48].

Trans/endobronchial cryobiopsy—can collect larger specimens than conventional biopsy to evaluate remodeling in the large and distal airway. Major complications of this procedure are linked to the larger samples taken, which increases the risk of major bleeding and pneumothorax. A recent study suggested that transbronchial lung cryobiopsy combined less invasiveness and higher capacity to provide enough samples for morphological analysis in asthma disease. Therefore, the authors indicated that it could be useful research in the pathobiology of asthma and severe asthma [49].

## 5. Indirect Assessment of Using Body Fluids

Bronchoalveolar lavage (BAL)—performed under flexible bronchoscopy, and it can be obtained at the same time as the endobronchial biopsy. It is used to study cellular composition and measure cytokine/chemokine levels in the distal airway and alveoli. Through the process of BAL, procollagen degradation and synthesis products, MMP, tissue inhibitor of metalloproteases (TIMP), and profibrotic cytokines can be quantified [45,50]. Limitations include Evaluation of remodeling is indirect, unable to discriminate between distal airway and alveoli, and limited to some soluble markers related to ECM production and degradation. BAL volume and withdrawn volume are inconsistent, leading to inconsistent dilution factor.

Induced sputum—a relatively noninvasive process and is commonly to evaluate central airway inflammation and levels of soluble remodeling-associated proteins: procollagen synthesis peptides, MMPs (MMP-2, MMP-9), TIMPs (TIMP-1, TIMP-2), cytokines, elastase, and α1-antitrypsin [51,52,53,54]. Limitations include that a fresh sputum sample without denaturation treatment is required. Inconsistent dilution factor (may contain saliva) and obtainment of the adequate specimen.

Exhaled breath condensate—a noninvasive but complicated method. It reflects the composition of the fluid lining the airway and successfully measures hydrogen peroxide, leukotrienes, prostaglandins, isoprostanes, nitric oxide-derived products, and hydrogen ions [55]. However, the measurement of mediators within exhaled breath condensate is not very reproducible, and the technique warrants improvement [56].

Blood—markers of collagen synthesis and degradation can be quantified in blood. Other markers, including MMP-9, TIMP-1, cytokines, eotaxin, and eosinophil cationic protein, have been measured in the plasma of asthma patients [57,58]. Limitations include that blood markers are not specific to airways and cannot determine if markers are from airways or other organs. Proteins synthesized in the lungs are not released in high enough quantities to be measured in the blood.

Urine testing—an unconventional way to evaluate airway remodeling. The study result, which was published by Priftis et al., demonstrated that urine tests could detect glycosaminoglycans, a component of ECM in urine samples of asthma patients [59]. However, this is not specific to airway remodeling.

## 6. Radiological Assessment

Computed tomography (CT)—allows the study of the airway lumen and wall dimensions without invasive process, which may be considered to evaluate airway remodeling in children and clinical studies [60,61]. It can identify lung, lobes, airway tree, and vascular tree and evaluate changes in remodeling after treatment as well as also can determine air trapping. Moreover, multi-detector CT can map disease probability, and 3-dimensional computer models can simulate and quantitate airflow patterns and assess changes [62].

Optical coherence tomography (OCT)—produces 2-dimensional images of the airway wall, which uses near-infrared light. For chronic obstructive pulmonary disease (COPD) patients, there is a strong correlation between OCT and CT measurements of average wall area and lumen area [63]. For asthmatics, who were evaluated by OCT, they had greater distension of the airways at a given pressure and had decreased lumen area compared with a control group [64]. Therefore, OCT can be used to monitor serial airway changes after a therapeutic intervention while avoiding cumulative radiation exposure [62].

Endobronchial ultrasound (EBUS)—performed with an ultrasonographic probe through the working channel of a fiberoptic bronchoscope. In the equine model, it can access airways as small as 4 mm in internal diameter and visualize multiple layers of the airway wall without the use of radiation [65], but cannot discriminate among different components of the airway wall such as smooth muscle bundles.

Positron emission tomography (PET)—able to measure pulmonary perfusion and ventilation when adding adequate isotope. According to previous studies, fluorodeoxyglucose (FDG)-PET was demonstrated as a good imaging biomarker of lung inflammation in asthmatic patients because neutrophils are the primary source of increased FDG uptake in the lungs. FDG-PET has the potential to be used to better understand asthma pathogenesis, phenotype differentiation, and response to anti-inflammatory treatment, but the cost is high [62].

Hyperpolarized (He, Xe) MRI—hyperpolarized noble gases are used as MRI contrast media for measuring pulmonary function biomarkers: lung ventilation, airway microstructure quantification, and gas exchange [62]. However, the high cost associated with specialized gas and equipment is the limitation.

## 7. Physiological Assessment

Pulmonary function test (PFT)—In clinical practice, a low post-bronchodilator FEV_1_/FVC ratio is one of the characteristics of airway remodeling. Furthermore, Ward et al. [66] found that a negative correlation has been observed between airway distensibility and RBM thickness in asthma. On the other hand, PFT also has limitations, which are the lack of the ability to assess the regional distribution of changes in local airway resistances and airway volume growth.

## 8. Conclusions

In conclusion, IgE is one of the major causes of the allergic inflammation process. IgE can interact directly and indirectly with effective inflammatory cells, such as dendritic cells, mast cells, eosinophils and, etc., as well as airway epithelial cells and ASMCs. Therefore, several Th2 mediated cytokines, IL-4, IL-5, IL-13, and remodeling mediators, IL-33, IL-25, TSLP, ET-1, MMP-9 and TGF-β, could be produced, promoting persistent airway inflammation. Through blocking IgE, it may break this vicious cycle to allow physicians to control patients’ asthma symptoms and to gain a better quality of life.

Omalizumab was the first and, for a long time, the only biologic drug registered for asthma therapy. It is able/capable of remarkably improving—when added to the standard antiasthma treatment—respiratory symptoms, quality of life, lung function and exacerbation rate [40,41,42,43,67]. Both placebo-controlled studies and real-world studies have unequivocally demonstrated the therapeutic effectiveness of omalizumab due to its capability of markedly blunting allergic airway inflammatory pathways [40,41,42]. These positive pharmacologic features are also associated with a very good profile of safety and tolerability. Moreover, current experimental evidence suggests that omalizumab cannot only interfere with the cellular and molecular mechanisms underlying airway remodeling but also modulate anti-viral immune activity [68,69,70], thus possibly behaving as a disease-modifying agent.

The gold standard assessment of airway remodeling evaluation is obtained by direct tissue assessment, surgical lung species, endobronchial biopsies, transbronchial biopsies and cryobiopsies, through flexible bronchoscopy. Although these assessment tools are standard, the main limitation is that few patients would be willing to undergo such an invasive evaluation process. Within the indirect analysis of BAL, blood, urine, induced sputum and exhaled breath condensate, the process is much easier than direct assessment tools, but not specific. Other alternative tools, including CT, EBUS/OCT, PET and MRI, can be easily studied, but the cost is high as well as related specialized gas and equipment are needed. Lastly, PFT is a regular study in clinical practice, and it can be used as screening tools. However, the correlation between PFT results and airway remodeling should be considered.

## Figures and Tables

**Table 1:** Summary of effects of immunoglobulin E (IgE) on airway remodeling.

First Author [ref.]	Year	Study Design	Effects on Airway Remodeling
Gounni [20]	2005	Experimental study In vitro Human ASMCs from atopic asthmatics *n* = 6	Human ASMCs express FcεRI∙ IL-4, IL-5, IL-13 and eotaxin released and human ASMCs contraction after IgE is stimulated
Redhu [21]	2013	Experimental study In vitro Human ASMCs	IgE-induced proliferation of human ASMCs via MAPK, Akt and STAT3 signaling pathways
Roth [22]	2013	Experimental study In vitro Human ASMCs Two groups (Allergic asthma and non-asthmatics, *n* = 8 each)	IgE increased human ASMCs proliferation and ECM and collagen deposition in a dose-dependent manner IgE effects were more prominent in asthmatic tissue Pre-incubation with omalizumab prevented all remodeling effects
Roth [23]	2015	Experimental study In vitro Human ASMCs Four groups (healthy controls, allergic asthma and nonallergic asthma, allergic without asthma, and *n* = 10 each)	IgE-induced proliferation of human ASMCs, collagen type-1 deposition, fibronectin Omalizumab inhibit these three effects of allergic serum in allergic asthma subjects, but no significant effect on serum IgE from healthy subjects or non- allergic asthma patients.
Fang [25]	2019	Experimental study In vitro Human ASMCs Two groups (asthmatics and control, and *n* = 5 each)	Non-immune IgE-stimulated ASMC remodeling by upregulating microRNA-21-5p, which downregulated PTEN and supported mTOR signaling.

Ai: luminal area at the right apical segmental bronchus; ACT: asthma control test; AQLQ: asthma quality of life questionnaire; AR: airway remodeling; ASMCs: airway smooth muscle cells; BAL: bronchoalveolar lavage; BSA: body surface area; COPD: chronic obstructive pulmonary disease; CT: computed tomography; IL: interleukin; IgE: immunoglobulin E; ET-1: endothelin-1; ECM: extracellular matrix; ECP: eosinophil cation protein; FEV1: forced expiratory volume in 1 s; FeNO: fractional exhaled nitric oxide; FVC: forced vital capacity; mTOR: mammalian target of rapamycin; MAPK: mitogen-activated protein kinase; MMP-9: metalloproteinase-9; PFT: pulmonary function test; PTEN: phosphatase and tensin homolog; RBM: reticular basement membrane; RCTs: randomized control trials; SAA: severe allergic asthma; STAT: signal transducer and activator of transcription; TNF: tumor necrosis factor; T/√BSA: wall thickness; WA: wall area.

**Table 2:** Summary of omalizumab for the effects on airway remodeling.

First Author [ref.]	Year	Study Design	Effects on Airway Remodeling
**Structure improvement**			
Riccio [28]	2012	Clinical study 11 severe allergic asthmatics 1 year omalizumab	Significant reduction in RBM thickness in bronchial biopsies Reduction of the number of infiltrating eosinophils (not significant)
Hoshino [29]	2012	Clinical study 30 severe allergic asthmatics Randomized 1:1 (omalizumab vs. conventional therapy for 16 weeks)	Omalizumab decreased WA/BSA, WA percentage and T/√BSA and increased Ai/BSA as assessed by CT Omalizumab decreased the percentage of sputum eosinophils and increased FEV1 and AQLQ scores Changes in FEV1 and sputum eosinophils correlated with changes in WA percentage
Mauri [30]	2014	Clinical study 8 severe allergic asthmatics 1 year omalizumab Proteomics of bronchial biopsies	Omalizumab downregulated bronchial smooth muscle proteins Among ECM proteins, galectin-3 correlated best with airway remodeling modulation by omalizumab
Tajiri [31]	2014	Clinical study 31 severe allergic asthmatics 48 weeks omalizumab	Omalizumab decreased WA percentage and the thickness and increased Ai and Ai/BSA as assessed by CT WA percentage changes significantly correlated with the decrease in FeNO50 levels and sputum eosinophils
Riccio [32]	2017	Clinical study 8 severe allergic asthmatics 36 months omalizumab	Omalizumab significantly reduced RBM thickness and related inflammatory proteins, including eosinophils, ASM proteins and periostin
Zastrzezynska [33]	2020	Clinical study 13 severe allergic asthmatics At least 12 months omalizumab	Omalizumab decreased basal lamina thickness and fibronectin deposit in airway submucosa Omalizumab reduced eosinophil counts in BAL, bronchial mucosa and blood (not significant)
Kardas [27]	2020	Review article regarding the proven effects of biologics in asthma on AR	The data available to date confirm with a high degree of probability only the beneficial role of omalizumab in reversing AR
**Regulation of Airway Remodeling Mediators**			
Djukanovic [36]	2004	RCT 45 mild to moderate asthma with sputum eosinophilia (With omalizumab, *n* = 22, Placebo, *n* = 23) 16 weeks	Reduced expression of eosinophils in both blood and sputum after omalizumab treatment
Massanari [34]	2010	Pool analysis of five RCTs	Blood eosinophils reduction after omalizumab treatment
Zietkowski [35]	2010	Clinical study 19 severe allergic asthmatics Two groups (With omalizumab, *n* = 9 vs. without Omalizumab, *n* = 10)	Omalizumab in patients with SAA results in decreased expression of ET-1 in the airways as well as reduced blood eosinophils, ECP and FeNO
Roth [44]	2010	Experimental study In vitro Human ASMCs Three groups (Allergic asthma, COPD and control, *n* = 6 each)	IgE stimulation increased IL-6, IL-8 and TNF-α mRNA synthesis and secretion by human ASMCs in all groups Omalizumab inhibited IgE-stimulated cytokine secretion in a dose-dependent fashion
Affara [39]	2015	Clinical study 50 severe allergic asthmatics 36 months omalizumab	Omalizumab reduced serum MMP-9 level and FeNO, in parallel, ACT and FEV1 improvement
Huang [37]	2019	Clinical study 23 severe allergic asthmatics More than 3 years omalizumab	The characteristics of omalizumab responder: Alarmin aggravated Type 2 high endotype, higher FeNO levels, more eosinophilic airway inflammation, poor asthma control and PFT Reduced alarmin level in bronchial tissue in both mRNA and protein level Reduced exacerbations, FeNO levels Improved ACT, FEV1 and FVC
**Pulmonary Function Improvement**			
Normansell [40]	2014	Cochrane systematic review 25 studies	A significant benefit regarding the change from baseline in FEV1% predicted for subcutaneous omalizumab versus placebo was observed
Alhossan [41]	2017	Real-world meta-analysis 25 observational studies, including 9213 patients across 32 countries	Compared with baseline, a significant improvement FEV1: 904% after 4–6 months of omalizumab treatment FEV1: 106% after 12 months of omalizumab treatment FEV1: 96% after 24 months of omalizumab treatment
MacDonald [42]	2019	Real-world meta-analysis 42 real-world evidence From 2008 to 2018	Compared with baseline, FEV_1_ improvement by an average of 85%­–124% between 5 and 32 months; by an average of 26% ≥ 36 months
Busse [43]	2020	Post hoc analysis of three RCTs, including pediatric and adolescent/adult population	Omalizumab may confer some protection against PFT decline among children, adolescent and adults, who experience asthma exacerbations while on therapy

Ai: luminal area at the right apical segmental bronchus; ACT: asthma control test; AQLQ: asthma quality of life questionnaire; AR: airway remodeling; ASMCs: airway smooth muscle cells; BAL: bronchoalveolar lavage; BSA: body surface area; COPD: chronic obstructive pulmonary disease; CT: computed tomography; IL: interleukin; IgE: immunoglobulin E; ET-1: endothelin-1; ECM: extracellular matrix; ECP: eosinophil cation protein; FEV1: forced expiratory volume in 1 s; FeNO: fractional exhaled nitric oxide; FVC: forced vital capacity; mTOR: mammalian target of rapamycin; MAPK: mitogen-activated protein kinase; MMP-9: metalloproteinase-9; PFT: pulmonary function test; PTEN: phosphatase and tensin homolog; RBM: reticular basement membrane; RCTs: randomized control trials; SAA: severe allergic asthma; STAT: signal transducer and activator of transcription; TNF: tumor necrosis factor; T/√BSA: wall thickness; WA: wall area.
